# Maintaining critical infrastructure resilience to natural hazards during the COVID-19 pandemic: hurricane preparations by US energy companies

**DOI:** 10.1186/s43065-020-00010-1

**Published:** 2020-08-06

**Authors:** Aaron Clark-Ginsberg, Ismael Arciniegas Rueda, Jonathon Monken, Jay Liu, Hong Chen

**Affiliations:** 1 RAND Corporation, 1200 S. Hayes St, Arlington, VA 22202 USA; 2 RAND Corporation, Arlington, VA USA; 3 PJM, Norristown, USA

**Keywords:** COVID-19, Critical infrastructure protection, Utilities, Sociotechnical resilience, Multi-hazard disasters, Hurricanes, Concurrent disaster, Energy resilience

## Abstract

The COVID-19 pandemic has the potential to compromise the ability of critical infrastructure utilities to respond to or mitigate natural hazards like wildfires and hurricanes. This article describes the ways that an energy organization, the regional transmission operator PJM, is preparing for hurricanes during the COVID-19 pandemic. PJM is using a combination of technological and organizational processes to prepare for hurricanes during the pandemic. Activities include the development of a third control room to increase redundancy and maintaining social distance at control center, investment in more resilient communications technology to maintain connectivity, and taking a holistic approach to identifying issues related to supply chain and fuel security. With this mix of organizational and technological processes, we argue that critical infrastructure resilience should be understood as a sociotechnical construct and identify several recommendations for improving resilience. The article has implications for policymakers working to maintain infrastructure resilience to natural hazards during the COVID-19 pandemic.

## Introduction

Maintaining critical infrastructure resilience to natural hazards during the novel coronavirus outbreak (COVID-19) is crucial yet challenging. The US Department of Homeland Security (DHS), understands critical infrastructures as infrastructure sectors such as water supply, public health, transportation, and energy that are considered essential to national security, economy security, public health or the safe functioning of a society [3]. While the COVID-19 virus itself has no impact on the physical architecture of these infrastructures, policymakers suggest that it appears to be straining the various organizational and social practices necessary for their operation. For instance, the US energy sector’s Electric Sector Coordinating Council has described how workers responsible for maintaining business continuity and reacting to natural hazards, the supply chains used to secure crucial equipment and supplies, and the mutual aid that utilities rely on, are being compromised [7]. Similarly, the lead US agency responsible for critical infrastructure security, the DHS’s Cybersecurity and Infrastructure Security Agency, recently described how it was “imperative” to maintain critical infrastructure during the pandemic and outlined several strategies for doing so [6].

This commentary describes how policymakers can maintain critical infrastructure resilience to natural hazards during the COVID-19 pandemic. Literature on multi-hazard events, including concurrent disasters and cascading crisis, indicates that, especially when vulnerabilities are shared, the confluence of multiple hazards can pose additional challenges compared to managing a single hazard alone [5, 17, 18]. Already under strain from the COVID-19 pandemic, there are real questions about how policymakers can keep critical infrastructure up and running during a natural hazard event.

To do so, the commentary focuses on the steps that one energy organization, the US regional electric transmission operator (RTO) PJM, is taking to ensuring critical infrastructure resilience during the upcoming hurricane season in light of the COVID-19 pandemic. A resilience orientation may be a necessary approach for maintaining continuity of operations during the confluence of the COVID-19 pandemic and a natural hazard event. Resilience generally refers to the ability of a system to resist, absorb, accommodate, recover, and transform in the face of a shock, stress, or other crisis [12]. Resilience has been growing as a policy solution for critical infrastructure [11], and for good reason: the increasing complexity and interconnections of infrastructure systems coupled with growing hazard uncertainties means that unavoidable complexity and coupling induced “normal accidents” [16] are becoming increasingly likely. By emphasizing the need to invest in mitigation and other forms of hazard prevention but also be prepared to react quickly and decisively to limit damages, recover, and learn from and adapt when disasters do occur, resilience seems to be an ideal paradigm for maintaining critical infrastructure in an increasingly hazardous world.

The electric grid is a useful infrastructure to focus on for this study because of its criticality and complexity. Energy is crucial for a functioning society: not only is the energy sector a critical infrastructure, but it is arguably the most critical infrastructure, with all other infrastructure dependent on energy systems [21]. Furthermore, the sheer scale and complexity of energy systems combined with their tight coupling makes maintaining energy resilience particularly challenging. Electric grids are dispersed ‘system of systems’: the North American electric grid is comprised of thousands of utilities that run the generation, transmission, and distribution systems across North America. Likewise, the synchronous grid of Continental Europe is operated by public and private entities and stretches across 24 countries to supply over 400 million customers with power. Interdependencies mean that energy grids could be vulnerable to cascading events. In Europe, natural hazards, cyberattacks, space weather, and other disasters have the potential to trigger widespread and far reaching system-level cascades [9]. In North America, these cascades have been observed as large grid failures such as the 2003 Northeast blackout, a cascading scenario triggered when a loss in system visibility meant that control center operators were not alerted to a failure in a transmission line. The cascading voltage collapse on the transmission system rippled across the Northeast US and southern Canada, causing a blackout covering 11 states and the Canadian province of Ontario that left an estimated 55 million people without power [24]. It is no wonder that the US electric grid is described as “the most complex machine ever made” [13].

As a regional transmission operator, PJM is at the heart of this complex machine. PJM is responsible for reliable power grid operations, efficient wholesale markets, and infrastructure development in 13 states and the District of Columbia. PJM’s footprint alone is comprised of more than 1300 generation facilities producing power and 84,000 miles of transmission lines delivering it across 243,417 mile^2^ of territory, and distribution assets that bring power to more than 65 million customers. PJM works with the owners and operators managing these assets to ensure reliability and resilience.

We identify and describe several strategies that PJM is taking to prepare for hurricane season in the light of the COVID-19 pandemic. These involve actions of PJM itself as well as support to owners and operators within PJM’s footprint and include developing a third redundant control room and working to invest in more resilient communications technology to maintain connectivity for essential operations. They also involve both technological and organizational processes: we therefore argue that infrastructure resilience to hurricanes and COVID-19, like other hazards, is a sociotechnical construct [1, 4, 8], with resilience dependent not just technology but also social and organizational factors. The commentary has implications for policymakers concerned with maintaining the resilience of large, interconnected and interdependent critical infrastructure similar to the electric grid such as water, communications, and oil and gas.

## PJM, COVID-19, and the 2020 hurricane season

Similar to general definitions of resilience [12], PJM understands energy resilience as the ability of the energy system to withstand and reduce the magnitude or duration of disruptive events [2]. A resilient energy infrastructure is an infrastructure that can withstand negative events with minimal loss of load and few disruptions to water, communications, transportation, and other critical infrastructure. Ensuring energy system resilience involves reducing the overall number of system disruptions and through mitigation activities like infrastructure hardening, and improving the speed of post-event response through activities like contingency planning. Since events are often difficult to predict and their impacts cannot always be anticipated, resilience also involves adaptation to a rapidly changing operational environment.

PJM has several measures in place to ensure energy resilience to hurricanes, many of which focus on improving response through quickly deploying crews and replacing essential grid components after hurricanes. To maintain a flow of energy, PJM maintains a reserve margin of generation assets to supply energy when other facilities are impacted and has plans in place for directing power flows in real-time from stable portions of the grid to impacted areas. To ensure it is able to balance energy loads, it has redundant control room facilities that staff can use if one is compromised. To get impacted areas up and running quickly, PJM works to reduce potential supply chain lags by pre-staging critical assets like extra high voltage transformers and other spare equipment, and has established plans designed to facilitate rapid response.

The COVID-19 pandemic may already be affecting PJM’s ability to respond to hurricanes in two important ways. First, the pandemic is impacting the supply chains that PJM relies on for equipment, especially equipment with long production lead times that is typically manufactured internationally. Having the right replacement equipment is crucial for mitigating damages and restoring power in a timely manner. The pandemic is impacting the ability of manufacturing companies to maintain personnel and raw material necessary for producing equipment, which is slowing down equipment production across the world. PJM and the utilities that it works with already have some spare equipment that can be used in in emergency, but excess margins are thin so PJM and utilities remain by in large reliant on just-in-time delivery for spares. A large hurricane could result in a sudden and dramatic increase in demand for replacement equipment beyond the current ability of supply chains. Second, the pandemic is creating personnel issues that have the potential to disrupt response capacity. The pandemic is reducing the available workforce and affecting the ability of utilities to implement typical response strategies, which involve putting people in close quarters and moving them long distances, e.g. close-proximity crew staging and transporting personnel outside the affected area to response sites. Maintaining control rooms is also particularly challenging: crucial for managing load in real time as disasters unfold, control room operators work together in close quarters, which increases chances for exposure, and are highly specialized, so would not be easy to replace if infected with coronavirus.

PJM is taking several steps to respond to these challenges. First, is limiting maintenance activities that might create outages to reduce the chances of large-scale outages. The spring season is typically the maintenance outage season, but PJM is working with generation and transmission owners to reschedule these planned outages to prevent them from potentially turning into larger disruptions. Second, to address equipment issues, PJM has issued guidance for utilities stating that utilities should “… not be reliant on another party or even the vendor for immediate spare support” [20] and recommends that they review their plans and check with vendors on delivery lead time in light of COVID-19.

Third, PJM working with utilities to help them modify their hurricane response staffing plans. Staff are following CDC and state agency COVID-19 workplace recommendations, utilizing appropriate personal protective equipment (PPE) and maintaining social distancing as the “new normal” at job sites. Specialized PPE, such as flame-resistant rated face masks, are available. Utilities are also adopting new telecommunication tools and cloud-based restoration plans and work orders to support telework for functions that were previously done by personnel on-site. PJM suspended its own staff travel in January and implemented company-wide work from home guidelines in early March.

PJM is also working to ensure control center continuity during the pandemic. To reduce staff risk of exposure, PJM suspended all in-person access to the control centers except for control room staff (including staffing real-time reliability engineers outside rather than within control rooms), has increased control center staff shift length to 12-h to reduce exposure during staff changeovers, and is performing outage studies remotely. PJM also converted its control room training simulator to a third control center to increase control room redundancy and reduce staff exposure. All three control rooms can now function together as a single virtual control room, and each facility is capable of operating the entire system independently if the others are compromised. As part of this, in mid-April PJM also sequestered a group of dispatchers to operate from the newly-created control room to ensure the availability of a full shift if the virus were to spread within PJM.

Response-based activities are only one part of COVID-19 and hurricane resilience; resilience is also being facilitated through mitigation activities focused on grid modernization and infrastructure hardening. PJM is using a standard and modularized design to modernize aging infrastructure, which makes having unique spare equipment on hand less necessary, reducing the impact of supply chain shortages and down time for repair and restoration. Substation automation at critical and remote sites integration with internet of things technologies reduces the need people to be physically present for real-time equipment condition diagnoses and reconfigurations, reducing impacts from workforce restrictions. New equipment is also more robust, meaning it can more easily stand up to hurricanes and other disasters.

Sensemaking is a crucial part of all of this. Sensemaking refers to the ability to see the ‘big picture’ by putting all the disjointed elements of information together, finding relationships between them, and assigning meaning to the scenario [25]. Unlike hurricanes and storms, which occur with relative regularity and can therefore often be managed in a somewhat ‘routine’ manner, sensemaking is particularly critical for novel threats with high degrees of uncertainty [25]. PJM’s sensemaking activities include conducting studies on specific risks within its infrastructure and engaging in collaboration with networks of other actors to determine broader trends and develop more general strategies. Some of PJM’s studies include stress-test studies of systems under extreme situations. PJM has developed and analyzed over 8000 different contingencies, such as gas pipeline disruptions that could impact the availability of fuel for generators, or the loss of multiple generation units and transmission facilities within a disaster impacted area. It also conducts cascade analyses to help understand how large cascading outages like the 2003 blackout might manifest and identify how they can be contained.

Sensemaking is partly also a networked property for PJM, and PJM maintains a close collaboration with a network of stakeholders to understand resilience related issues. PJM has a long history of collaborating with many organizations to understand how to support resilience. These include generation, transmission, and distribution utilities; other RTOs and independent system operators; the industry body for the bulk electric system, the North American Electric Corporation; the Electricity Subsector Coordinating Council (ESSC); and the North American Transmission Forum. For the COVID-19 pandemic, PJM is leveraging this network to learn from utilities that have had to respond to extreme weather during the outbreak, such as lessons that the ESCC has collected following the spring tornado season in the southeast and other small-scale events impacting the grid [7]. It has also come together with other organizations within its network to rapidly develop “crowdsourced” pandemic guidelines based on their collective experiences and knowledge. This resulted in several resource guides, such as a guide on natural hazard and COVID-19 response published by the IEEE Power and Energy Society designed to be shared widely across the industry, be adapted to company-specific needs, and amended as circumstances shift during and the pandemic evolves [15].

## Discussion

Policymakers working to maintain critical infrastructure resilience to natural hazards during the COVID-19 pandemic can take several lessons from PJM’s experience preparing for the hurricane season and use them to enhance resilience. First, policymakers working towards natural hazard resilience may need to assess and intervene on the resilience of systems not from a perspective of technology alone, nor from a perspective of organizational activities, but from a sociotechnical perspective that emphasizes both the technological and organizational dimensions of utility resilience. The ability of PJM to adapt its hurricane response systems for the COVID-19 pandemic appears dependent on its physical infrastructure such as control centers, grid modernization technologies, and technologies that allow for remote work, as well as organizational changes including changing shift lengths, work habits, sequestering workers to reduce exposure—all of which is buffered and supported by an organizational focus on sensemaking and adaptation. Indeed, the RTO’s response ability relies largely on organizational activities, including emergency response guidelines, situation awareness, and transmission operator field responses. Its resilience is a function of natural uncertainties and human decisions through the event, where teamwork and collaborations are core control factors shaping grid resilience.

Second, the experience of PJM also indicates that vulnerability and resilience of critical infrastructure to concurrent disasters that include a new hazard such as COVID-19 is partially shaped by its previous vulnerability and resilience activities and processes. Vulnerabilities shaping the potential for hurricane and COVID-19 to interact and cascade, such as a reliance on globalized just-in-time supply chains and on highly specialized staff that are difficult to replace, exist before COVID-19 and influence hurricane risk. Likewise, many of PJM’s systems for other hazards – like the preparedness plans it had in place, its methods of working with other utilities and policymakers for information sharing and adaptiveness, and its multiple redundant technological systems – are being harnessed for a concurrent COVID-19 pandemic and hurricane season. Further, the institutional culture of reliability appears agnostic to hazard compromising reliability, with PJM staff and leadership as concerned with the impacts of COVID-19 compromising its operations as hurricanes and storms. PJM’s ability to address duel threats of hurricanes and COVID-19 is predicated on strong existing systems for mitigation, preparedness, and adaptation, supported by sensemaking. Resilience to one threat is not created in a vacuum; instead broader hazard-agnostic processes can be leveraged. Thus, policymakers may need to leverage their existing multi-hazard activities for hurricane preparedness, particularly to reduce vulnerabilities that appear to link these hazards and spur on cascading crises. We therefore reiterate calls made by Pescaroli et al. [19] to place vulnerability reduction front and center as part of addressing cascading crises and supporting critical infrastructure resilience. Given standards and regulations play a crucial role in shaping critical infrastructure vulnerability and resilience [4, 10], as part of this process relevant critical infrastructure benchmarks and standards – such as NFPA 1600:2019, ISO 22301:2019, NERC’s Reliability Standards, and Section 1235(b) of the Disaster Recovery Reform Act of 2018 – may need to be refined to move away from focusing on single hazards to emphasize vulnerability reduction and resilience to cascading crises.

Third, PJM’s experience in resilience is not just the sole outcome of PJM but rather the product of a broad network of activities. This is unsurprising given high reliability theorists and other organizational scholars have identified the networked nature of certain critical infrastructure as not just a factor leading to disaster cascades, but as a crucial variable for maintaining ‘networked reliability’ [22, 23]. What is surprising here is just how much of the network benefits were not just for sharing equipment and other resources and responding to disasters in real time, but were focused on epistemic networks of people working together to collectively to understand how to prepare for hurricanes during the COVID-19 pandemic. Such epistemic networks, seen in PJM’s engagement with other utilities and sector-level coordinating groups, appear crucial for adaptiveness and sensemaking for novel, emergent threats.

Although this case study sheds some light on how utilities are preparing for natural hazards during the COVID-19 crisis, there are also core research questions that still need to be understood. First, consider that PJM has taken steps to mitigate the impact of COVID-19 on the global supply chain, however this epidemic has identified vulnerabilities on the US Bulk Power system that should be corrected. Thus, an area of research would be to identify alternatives to the procurement of key electric equipment considering that COVID-19 duration is unknown, it has a global scope and it may take several years to be resolved. Since reliance on just-in-time delivery mechanisms and global supply chains also contributes to vulnerability to hurricanes and other natural hazards, identifying alternative procurement mechanisms could also reduce natural hazard risk more generally and prevent cascades between hazards. Next, consider that COVID-19 is just one more on a list of related viruses such as SARS and MERS, it is likely that similar events would happen in the future. Thus, one recommendation is to expand the cascade analysis event tools to also include pandemics impact of load suppression and increased remote work on rapidly identifying and acting on grid dispatching. As reported by NERC, bulk power system organizations have been responding appropriately [14], however what would be the impact on accumulation of stresses on the system sociotechnical capabilities needs to be studied. Given the parallels between the US electric grid and other critical infrastructure inside and outside of the United States, answering these questions could help improve critical infrastructure resilience to concurrent crisis and disasters broadly, across multiple infrastructures and geographic locations.

## Conclusion

This commentary overviewed how policymakers can maintain critical infrastructure resilience to natural hazards during the COVID-19 pandemic by describing the activities that PJM was undertaking to prepare for the upcoming hurricane season. PJM is taking several activities that involve both organizational and technological changes, working with other electric grid stakeholders and supported by an organizational and institutional culture that promotes sensemaking and values resilience. We therefore argue that policymakers looking to maintain critical infrastructure to natural hazards during COVID-19 pandemic should 1) approach resilience from a sociotechnical perspective, 2) enhance existing resilience processes that may be hazard agnostic, particularly those that reduce vulnerability common to multiple threats, and 3) leverage network of stakeholders to share information, support adaptiveness, and coordinate physical resources. Although these actions are going far to ensure resilience during the COVID-19 pandemic, serious research must still be undertaken to prepare for the hazard season.

## Data Availability

Not applicable.

## Abbreviations

DHS: Department of Homeland Security
ESSC: Electricity Subsector Coordinating Council
IEEE: Institute of Electrical and Electronics Engineers
PPE: Personal protective equipment
RTO: Regional transmission operator
SARS: Severe Acute Respiratory Syndrome
MERS: Middle East Respiratory Syndrome
